# Epidemiological characteristics of human coronaviruses among populations with acute respiratory infections: surveillance data from jing'an district, Shanghai, 2024–2025

**DOI:** 10.1016/j.pmedr.2026.103401

**Published:** 2026-02-04

**Authors:** Qi Shen, Shuiping Lu, Qingyuan Xu, Mengting Tang, Yi Li, Bing Shen, Mingyi Cai, Chenglong Xiong

**Affiliations:** a Shanghai Jing'an District Center for Disease Control and Prevention (Shanghai Jing'an District Health Supervision Institute), Shanghai 200072, China; b School of Public Health, Fudan University, Key Lab of Public Health Safety, Ministry of Education, Shanghai 200433, China

**Keywords:** Human coronaviruses, Epidemiological characteristics, Acute respiratory infection, Multipathogen surveillance, Co-infection

## Abstract

**Objective:**

To investigate the epidemiological characteristics of common human coronaviruses (HCoVs) among populations with acute respiratory infections in the post-pandemic period.

**Methods:**

Detection data from the 2024–2025 comprehensive acute respiratory infection surveillance in Jing'an District, Shanghai, were analyzed using descriptive epidemiological methods to assess HCoVs' detection rates, demographic and seasonal patterns, co-infections, and their trends relative to influenza and other common respiratory viruses.

**Results:**

A total of 4758 acute respiratory infection cases were included, with an overall detection rate of HCoVs of 4.9% (232/4758). HCoV-NL63 (53.9%, 125/232) and HCoV-OC43 (22.8%, 53/232) were the predominant subtypes. Children under 14 years and adults over 65 years constituted susceptible populations. Different subtypes peaking at different times. HCoV-NL63 was predominant during summer and autumn; HCoV-OC43 in autumn and winter; HCoV-HKU1 in winter and spring; and HCoV-229E was sporadically year-round. Nearly 40% of the detections involved co-infections (38.8%, 90/232), and HCoVs showed alternating and co-circulating trends with other common respiratory viruses.

**Conclusions:**

HCoVs exhibit subtype-specific predominance in different seasons, with frequent co-infections and a substantial burden in children and the elderly, supporting the need for integrated surveillance and targeted protection.

## Highlights

• Human coronaviruses were detected in 4.9% of 4758 acute respiratory infection cases.
• Human coronavirus NL63 and OC43 were the predominant subtypes.
• Children under fourteen and adults over sixty-five were most susceptible.
• Human coronaviruses had subtype-specific seasonal peaks and frequent co-infections.
• Emphasizing ongoing surveillance of multiple respiratory pathogens.

## 1 Introduction

Human coronaviruses (HCoVs), including human coronavirus NL63 (HCoV-NL63), human coronavirus 229E (HCoV-229E), human coronavirus OC43 (HCoV-OC43), and human coronavirus HKU1 (HCoV-HKU1), are common pathogens responsible for acute respiratory infection (ARI), accounting for approximately 15–30% of such infections annually (Audi et al., 2020; Fehr and Perlman, 2015). In most cases, HCoV infections manifest as mild to moderate upper respiratory tract symptoms; However, in infants, the elderly, and immunocompromised individuals, they may progress to lower respiratory tract infections or even severe disease (Dijkman et al., 2012; Kesheh et al., 2022; Ou et al., 2017). Under previous circulation patterns, HCoVs exhibited endemic circulation with distinct seasonal fluctuations, and their epidemic peaks varied among subtypes. Moreover, infection rates differed across study regions and populations (Shah et al., 2022; Wilson et al., 2024). As important background respiratory viruses, the continuous circulation of HCoVs not only increases the overall burden on individual health but may also exert additional pressure on public health systems when co-circulating with influenza viruses, respiratory syncytial virus (RSV), and severe acute respiratory syndrome coronavirus-2 (SARS-CoV-2) (Ogimi et al., 2020; WHO, 2024).

Several systematic reviews and multicenter studies on the epidemiology of HCoVs have been conducted internationally (Huang et al., 2025; Wilson et al., 2024). However, epidemiological data from different regions of China remain relatively limited. Under the context of normalized COVID-19 prevention and control measures, altered population immunity levels, and the gradual restoration of social order, the regional circulation patterns of HCoVs in the post-pandemic period have not yet been sufficiently characterized. In particular, it remains unclear whether the epidemiological characteristics of HCoVs in the post-pandemic era have returned to their pre-pandemic endemic patterns or show distinct differences, underscoring the need for further investigation based on continuous local surveillance data. Therefore, this study utilized the 2024–2025 comprehensive acute respiratory infection surveillance data in Jing'an District, Shanghai, to systematically analyze the detection patterns, seasonal distribution, population susceptibility characteristics, and co-infection profiles of HCoVs. Moreover, we compared the epidemiological trends of HCoVs with those of other common respiratory viruses, including influenza viruses, RSV, SARS-CoV-2, human metapneumovirus (HMPV), and rhinovirus. This study aims to provide region-specific epidemiological insights into the transmission patterns of HCoVs in the post-pandemic era, thereby contributing complementary evidence to the understanding of HCoV epidemiology and offering an evidence base for clinical management, public health prevention and control, and future research on respiratory pathogens.

## 2 Methods

### 2.1 Study design and population

Using data from the 2024–2025 comprehensive acute respiratory infection (ARI) surveillance data in Jing'an District, Shanghai, we analyzed the circulation patterns of four common human coronaviruses (HCoV-OC43, HCoV-229E, HCoV-NL63, and HCoV-HKU1) during this period. Four sentinel hospitals were included in Jing'an District, Shanghai: Shanghai Children's Hospital, Huashan Hospital Affiliated to Fudan University, Shanghai Jing'an District Central Hospital, and Shanghai Zhabei Central Hospital. Within this ARI surveillance framework, patients were enrolled based on the case definitions for influenza-like illness (ILI) and severe acute respiratory infection (SARI), which represent outpatient and inpatient subsets of ARI, respectively. ARI was defined as follows: (1) acute onset within 10 days; (2) at least one of the following symptoms/signs: sore throat, cough, expectoration, nasal congestion, runny nose, chest pain, tachypnea, and abnormal pulmonary breath sounds; and (3) with or without fever. ILI was defined as an acute onset of illness with fever (body temperature ≥ 38 °C) accompanied by either cough or sore throat. SARI was defined as a hospitalized patient who, at the time of admission or within 48 h after admission, presented with an acute onset of illness, a history of fever (measured temperature ≥ 38 °C), cough, and symptom onset within the preceding 10 days. All sentinel hospitals registered and reported ILI and SARI cases. The study protocol was approved by the Medical Ethics Committee of Shanghai Jing'an District Central Hospital. Individual informed consent was waived as only anonymized data were used.

### 2.2 Measures

#### 2.2.1 Sample collection

ILI cases were identified, registered, and sampled in outpatient and emergency settings, including general internal medicine clinics, pediatric clinics, and fever clinics at the sentinel hospitals. SARI cases were identified, registered, and sampled in inpatient settings, including departments of respiratory medicine, pediatrics, infectious disease wards, intensive care units, and specialized emergency intensive care units. At each sentinel hospital, 10–40 ILI specimens were collected weekly, with an annual average of approximately 20 specimens per week. For SARI surveillance, 5–15 specimens were collected weekly at each hospital, with an annual average of approximately 10 specimens per week. Respiratory specimens included throat swabs, nasal swabs, nasopharyngeal swabs, or sputum samples.

#### 2.2.2 Specimen handling and transportation

For each enrolled patient, two specimens of the same type were collected simultaneously. One specimen (without antibiotics, non-inactivated) was used for bacterial detection and transported at room temperature, while the other (with antibiotics, non-inactivated) was used for viral detection and stored at 2–8 °C, avoiding repeated freeze-thaw cycles. All specimens were transported to the laboratory within 48 h after collection, and multiplex respiratory pathogen testing was performed within 48 h of sample receipt.

#### 2.2.3 Laboratory testing

According to the Shanghai Municipal Sentinel Surveillance Protocol for Acute Respiratory Infectious Diseases, all specimens were subjected to multiplex pathogen testing. Viral nucleic acids were extracted using a magnetic bead-based viral nucleic acid extraction kit (Jiangsu Shuoshi Biotechnology Co., Ltd., China), and bacterial DNA was extracted using a magnetic bead-based bacterial DNA extraction kit (Jiangsu Shuoshi Biotechnology Co., Ltd., China). Nucleic acid extraction, enrichment, and purification were performed with the fully automated nucleic acid extraction instrument SMPE-960 (Jiangsu Shuoshi Biotechnology Co., Ltd., China). The processed nucleic acid products were subsequently analyzed by the real-time quantitative PCR instrument LightCycler® 480 II (F. Hoffmann-La Roche Ltd., Basel, Switzerland). The multiplex real-time PCR kits used in this study were produced by Beijing Zhuocheng Huisheng Biotechnology Co., Ltd., China. The Real-Time PCR Diagnostic Kit Rapid Detection of multiple pathogens of national acute respiratory infectious diseases (SMS-D404AAYF-C-10 T-01 K) targeted SARS-CoV-2, influenza viruses, RSV, HCoVs, HMPV, rhinovirus, adenovirus, human parainfluenza viruses, human bocavirus, and enteroviruses. The Multiplex Real-Time PCR Diagnostic Kit for Rapid Detection of Pathogenic Respiratory syndrome (SMS-D424PQH-O2 (LS)) detected *Group A Streptococcus*, *Bordetella pertussis*, *Streptococcus pneumoniae*, *Haemophilus influenzae*, *Legionella spp.*, *Klebsiella pneumoniae*, *Mycoplasma pneumoniae*, *Aspergillus spp.*, *Cryptococcus spp.*, *Chlamydia psittaci*, and *Chlamydia pneumoniae*. Both multiplex real-time PCR kits contained pathogen-specific primers and fluorescent probes targeting the corresponding genes. Detection was performed by collecting fluorescence signals generated during PCR amplification, allowing qualitative detection of nucleic acids from the listed respiratory pathogens.

### 2.3 Statistical analysis

Descriptive epidemiological methods were applied to examine the detection rates of HCoVs, along with their distributions across age, sex, and seasonal patterns. Pearson's chi-square or Fisher's exact tests were used to compare categorical variables between groups. A two-sided *P* value <0.05 was considered statistically significant. All the statistical analyses were conducted using IBM SPSS Statistics® for Windows version 25.0. In addition, co-infections of HCoVs with other respiratory pathogens (specimens positive for two or more pathogens), were analyzed. In combination with overall detection and co-infection patterns, the epidemiological trends of HCoVs were then compared with those of other common respiratory viruses. Data analysis was conducted using IBM SPSS Statistics® for Windows version 25.0 and Microsoft® Excel® 2021. Figures were generated using Python version 3.11.9.

## 3 Results

### 3.1 Overall respiratory surveillance and detection of HCoVs

From January 1, 2024, to December 31, 2025, a total of 4758 acute respiratory infection (ARI) surveillance cases were collected, including 3977 influenza-like illness (ILI) cases and 781 severe acute respiratory infection (SARI) cases (Table 1). Among these, 2824 (59.4%) were positive for at least one monitored pathogen (indicating co-infections involving multiple pathogens), while the remaining 1934 were negative for pathogens. Of the total cases, 2403 (50.5%, 2403/4758) were positive for viruses, 707 (14.9%, 707/4758) for bacteria, 83 (1.7%, 83/4758) for *Mycoplasma pneumoniae*, eight (0.2%, 8/4758) for *Chlamydia pneumoniae*, six (0.1%, 6/4758) for *Aspergillus spp.*, and one (0.02%, 1/4758) for *Chlamydia psittaci*; *Cryptococcus spp.* was not detected (Table 1). Among all monitored samples, the top three viruses were influenza viruses, SARS-CoV-2, and rhinovirus, while the top three bacteria were *Haemophilus influenzae*, *Streptococcus pneumoniae*, and *Klebsiella pneumoniae* (Table 1 and S1 Fig). Among ILI cases, the top three viruses were influenza virus, SARS-CoV-2, and rhinovirus, while the top three bacteria were *Haemophilus influenzae*, *Streptococcus pneumoniae*, and *Mycoplasma pneumoniae* (Table 1). Among SARI cases, the top three viruses were rhinovirus, SARS-CoV-2, and HCoVs, while the top three bacteria were *Klebsiella pneumoniae*, *Haemophilus influenzae*, and *Streptococcus pneumoniae* (Table 1).

**Table 1:** Overview of respiratory pathogen detections in the 2024–2025 comprehensive acute respiratory infection surveillance in Jing'an District, Shanghai.

Pathogens	All (*N* = 4758 cases)		ILI (*N* = 3977 cases)		SARI (*N* = 781 cases)	
	Frequency (n)^a^	Percent (%)^b^	Frequency (n)	Percent (%)	Frequency (n)	Percent (%)
SARS-CoV-2	524	11.0	494	12.4	30	3.8
Influenza viruses (flu)	646	13.6	624	15.7	22	2.8
fluA	529	11.1	509	12.8	20	2.6
A(H3N2)	219	4.6	212	5.3	7	0.9
A(H1N1)	310	6.5	297	7.5	13	1.7
fluB	117	2.5	115	2.9	2	0.3
Respiratory syncytial viruses (RSV)	93	2.0	86	2.2	7	0.9
RSV-A	50	1.1	50	1.3	0	0.0
RSV-B	43	0.9	36	0.9	7	0.9
Adenovirus	159	3.3	155	3.9	4	0.5
Human metapneumovirus	110	2.3	98	2.5	12	1.5
Human parainfluenza viruses (HPIV)	249	5.2	244	6.1	5	0.6
HPIV1	78	1.6	77	1.9	1	0.1
HPIV2	77	1.6	77	1.9	0	0.0
HPIV3	74	1.6	71	1.8	3	0.4
HPIV4	21	0.4	20	0.5	1	0.1
Human coronaviruses (HCoVs)	232	4.9	209	5.3	23	2.9
HCoV-229E	20	0.4	17	0.4	3	0.4
HCoV-NL63	125	2.6	118	3.0	7	0.9
HCoV-OC43	53	1.1	46	1.2	7	0.9
HCoV-HKU1	40	0.8	34	0.9	6	0.8
Human bocavirus	23	0.5	21	0.5	2	0.3
Rhinovirus	464	9.8	427	10.7	37	4.7
Enteroviruses	103	2.2	96	2.4	7	0.9
*Group A Streptococcus*	27	0.6	26	0.7	1	0.1
*Bordetella pertussis*	3	0.1	3	0.1	0	0.0
*Streptococcus pneumoniae*	266	5.6	256	6.4	10	1.3
*Haemophilus influenzae*	298	6.3	284	7.1	14	1.8
*Legionella spp.*	1	0.0	1	0.0	0	0.0
*Klebsiella pneumoniae*	193	4.1	72	1.8	121	15.5
*Aspergillus spp.*	6	0.1	1	0.0	5	0.6
*Cryptococcus spp.*	0	0.0	0	0.0	0	0.0
*Mycoplasma pneumoniae*	83	1.7	81	2.0	2	0.3
*Chlamydia psittaci*	1	0.0	0	0.0	1	0.1
*Chlamydia pneumoniae*	8	0.2	8	0.2	0	0.0

a The frequency of each pathogen may include both the samples with single infection and those with co-infections, and their total number is larger than the sum of samples with single and co-infections.

b Percent of samples is the frequency of samples detected as positive divided by the total number of samples (n/N).

232 HCoV-positive samples were detected, accounting for 4.9% (232/4758). Among these, 209 positive samples were obtained from ILI cases, while the remaining 23 positive samples were obtained from SARI cases (Table 2). The age range of the HCoV-positive patients spanned from one to ninety-two years. HCoV-NL63 was the most frequently detected strain with 125 cases, followed by HCoV-OC43 (53 cases), HCoV-HKU1 (40 cases), and HCoV-229E (20 cases) (Table 2). Detailed information is presented in Table 2.

**Table 2:** The epidemiological characteristics of human coronaviruses in the 2024–2025 comprehensive acute respiratory infection surveillance in Jing'an District, Shanghai.

Variables	Total (N = 4758)	HCoVs				
		HCoVs (+)^a^ (*N* = 232)	HCoV-229E (*N* = 20)	HCoV-NL63 (*N* = 125)	HCoV-OC43 (*N* = 53)	HCoV-HKU1 (*N* = 40)
Period						
2024	2008	85 (36.6%^b^)	6 (30.0%)	43 (34.4%)	28 (52.8%)	11 (27.5%)
2025	2750	147 (63.4%)	14 (70.0%)	82 (65.6%)	25 (47.2%)	29 (72.5%)
Cases						
ILI	3977	209 (90.1%)	17 (85.0%)	118 (94.4%)	46 (86.8%)	34 (85.0%)
SARI	781	23 (9.9%)	3 (15.0%)	7 (5.6%)	7 (13.2%)	6 (15.0%)
Gender						
Male	2228	91 (39.2%)	9 (45.0%)	49 (39.2%)	21 (39.6%)	15 (37.5%)
Female	2530	141 (60.8%)	11 (55.0%)	76 (60.8%)	32 (60.4%)	25 (62.5%)
Age groups, year						
0–4	562	51 (22.0%)	4 (20.0%)	33 (26.4%)	12 (22.6%)	5 (12.5%)
5–14	1481	82 (35.3%)	5 (25.0%)	59 (47.2%)	5 (9.4%)	15 (37.5%)
15–24	247	9 (3.9%)	0 (0.0%)	4 (3.2%)	2 (3.8%)	4 (10.0%)
25–44	1116	46 (19.8%)	7 (35.0%)	17 (13.6%)	15 (28.3%)	7 (17.5%)
45–64	513	15 (6.5%)	1 (5.0%)	6 (4.8%)	5 (9.4%)	3 (7.5%)
≥65	839	29 (12.5%)	3 (15.0%)	6 (4.8%)	14 (26.4%)	6 (15.0%)
Season						
Spring	1164	30 (12.9%)	2 (10.0%)	2 (1.6%)	8 (15.1%)	19 (47.5%)
Summer	1203	68 (29.3%)	7 (35.0%)	52 (41.6%)	7 (13.2%)	3 (7.5%)
Autumn	1256	99 (42.7%)	7 (35.0%)	68 (54.4%)	25 (47.2%)	2 (5.0%)
Winter	1135	35 (15.1%)	4 (20.0%)	3 (2.4%)	13 (24.5%)	16 (40.0%)

a HCoVs (+) indicates cases that tested positive for human coronaviruses.

b Percentages shown represent the proportion of cases within each category among HCoV-positive samples.

### 3.2 Epidemiological characteristics of HCoVs

**Fig. 1:**
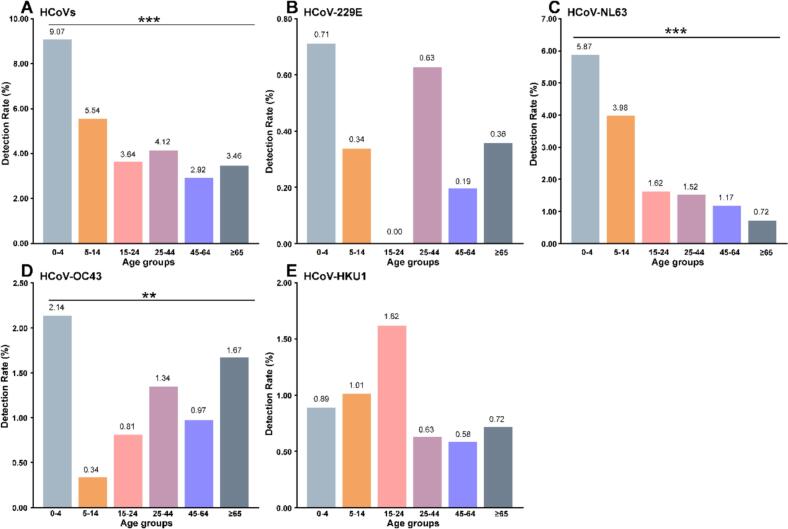
Human coronavirus infections by age in the 2024–2025 comprehensive acute respiratory infection surveillance in Jing'an District, Shanghai. A. HCoVs infections by age. B. HCoV-229E infections by age. C. HCoV-NL63 infections by age. D. HCoV-OC43 infections by age. E. HCoV-HKU1 infections by age. The star indicates a significant difference between the detection rates according to Pearson's chi-square test or Fisher's exact test. **: *P* < 0.01;***: *P* < 0.001.

HCoV infections exhibited marked age-related differences. The overall HCoVs detection rates differed significantly among age groups (χ^2^ = 32.79, *P* < 0.001) (S1 Table and Fig. 1). The highest HCoVs detection rate was observed in the 0–4 age group (9.1%, 51/562), followed by the 5–14 age group (5.5%, 82/1481). Both rates exceed the overall detection rate (4.9%, 232/4758) (S1 Table and Fig. 1A). In the remaining age groups, detection rates ranged from 2.9% to 4.1%, with the lowest rate observed among individuals aged 45–64 years (2.9%, 15/513). A slightly higher detection rate was observed in individuals aged ≥65 years (3.5%, 29/839) compared with the middle-aged group (S1 Table and Fig. 1A).

The distribution of four subtypes varied significantly across age groups. Significant age-related differences were observed for HCoV-NL63 (χ^2^ = 56.33, P < 0.001) and HCoV-OC43 (Fisher's exact test, *P* = 0.002), whereas no significant age-associated differences were identified for HCoV-229E or HCoV-HKU1 (Fisher's exact test, *P* = 0.62 and P = 0.62, respectively) (S1 Table and Fig. 1). The detection rates of HCoV-229E were consistently low across all age groups, ranging from 0.0% to 0.7%, with no evident age-specific clustering (S1 Table and Fig. 1B). HCoV-NL63 predominated in the 0–4 and 5–14 age groups, accounting for 64.7% (33/51) and 72.0% (59/82) of the relative groups, with detection rates of 5.9% (33/562) and 4.0% (59/1481), respectively, markedly higher than in other groups (S1 Table and Fig. 1C). HCoV-OC43 accounted for 23.5% (12/51) and co-circulated with HCoV-NL63 in the 0–4 age group, with a detection rate of 2.1% (12/562); HCoV-OC43 was the predominant subtype among older adults, accounting for 48.3% (14/29) in individuals aged ≥65 years, with a detection rate of 1.7% (14/839) (S1 Table and Fig. 1D). The detection rates of HCoV-HKU1 were low across all age groups. The highest detection rate was observed in individuals aged 15–24 years (1.6%, 4/247), whereas detection rates in the remaining age groups were below 1.1%, with no clear age-specific clustering observed (S1 Table and Fig. 1E).

Moreover, although a statistically significant difference in overall HCoVs positivity was observed between genders (χ^2^ = 5.66, *P* = 0.02), no significant gender differences were identified for infections caused by any of the four HCoV subtypes (all *P* > 0.05) (S1 Table).

**Fig. 2:**
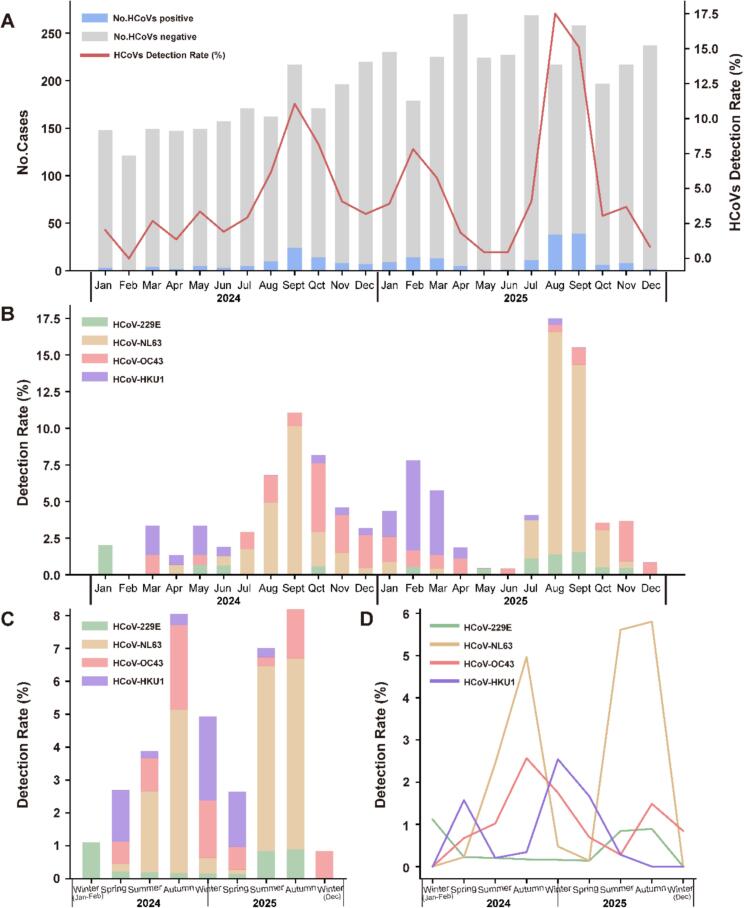
The prevalence and temporal distribution of human coronaviruses in the 2024–2025 comprehensive acute respiratory infection surveillance in Jing'an District, Shanghai. A. The number of HCoV-positive cases and detection rates among patients with acute respiratory infections by month. B. The detection rates of four subtypes by month. C. The detection rates of HCoVs by season. D. The detection rates of four subtypes by season. In panels C and D, the first “Winter” on the x-axis represents January–February, while the last “Winter” corresponds to December.

HCoVs were detected year-round and showed marked seasonal fluctuations, peaking in late summer and early autumn (August–October), with elevated activity also observed in winter (S2 Table and Fig. 2A). Among the four subtypes, HCoV-NL63 was the most predominant, accounting for 53.9% (125/232) of HCoV-positive cases (Table 2). HCoV-NL63 infections were mainly observed at the transition from summer to autumn, with the highest detection rates in August and September (Table 2, S2-S3 Tables and Fig. 2B-2D). HCoV-OC43 activity increased during autumn and winter (S3 Table and Fig. 2B-2D). HCoV-HKU1 was mainly detected in winter and spring, with the highest detection rate observed in winter (December 2024 to February 2025, 2.5%). The detection during spring were 1.6% in 2024 and 1.7% in 2025, respectively. (S3 Table and Fig. 2B-2D). However, HCoV-229E was sporadically detected throughout the year, showing low detection rates ranging from 0.0% to 1.1% and no distinct seasonal pattern (S3 Table and Fig. 2B-2D).

### 3.3 HCoVs single and Co-infections

**Fig. 3:**
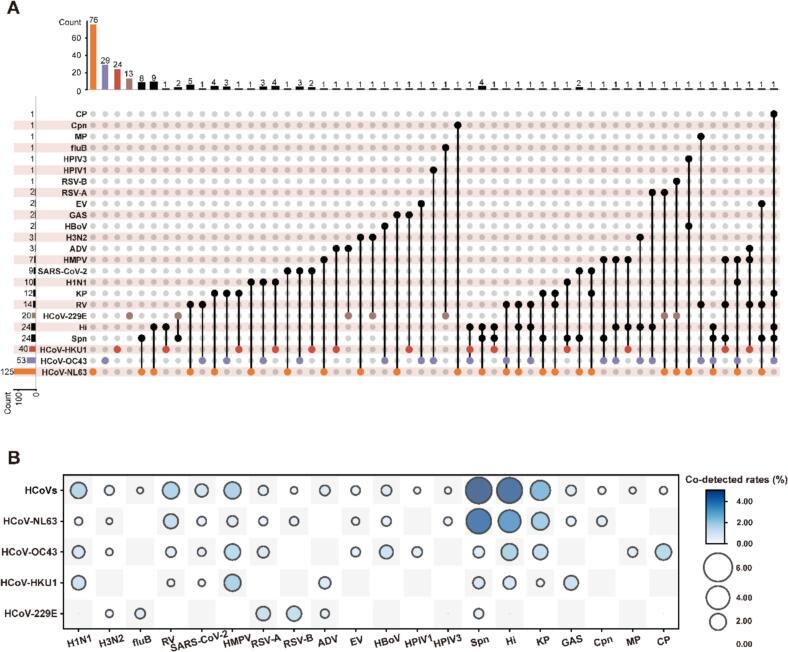
Human coronaviruses single and co-infection in the 2024–2025 comprehensive acute respiratory infection surveillance in Jing'an District, Shanghai. A. HCoVs single and co-infection pattern in patients with acute respiratory infections. A dot represents the presence of a pathogen infection, and black straight lines linking multiple dots indicate co-infections among pathogens. The horizontal bars represent the detection counts of each pathogen, while the vertical bars indicate the detection counts of each infection pattern (single or co-infection). B. Co-detected pattern of HCoVs and other respiratory pathogens in patients with acute respiratory infections. Co-detected rates were calculated pairwise. For pathogens “X” and “Y”, the numerator was the number of patients in whom “X” and “Y” were co-detected. The denominator was the total number of patients tested “X” and “Y”. The Bigger and darker circles indicate higher co-detected rates between the two pathogens. Several respiratory pathogens were included: human coronaviruses (HCoVs), human coronavirus NL63 (HCoV-NL63), human coronavirus 229E (HCoV-229E), human coronavirus OC43 (HCoV-OC43), human coronavirus HKU1 (HCoV-HKU1), rhinovirus (RV), adenovirus (ADV), enterovirus (EV), human metapneumovirus (HMPV), respiratory syncytial virus A (RSV-A), respiratory syncytial virus B (RSV-B), Severe Acute Respiratory Syndrome Coronavirus-2 (SARS-CoV-2), influenza A virus (H1N1), influenza A virus (H3N2), influenza B virus (fluB), human parainfluenza virus 1 (HPIV1), human parainfluenza virus 3 (HPIV3), human bocavirus (HBoV), *Streptococcus pneumoniae* (Spn), *Haemophilus influenzae* (Hi), *Klebsiella pneumoniae* (KP), *Group A Streptococcus* (GAS), *Mycoplasma pneumoniae* (MP), *Chlamydia psittaci* (CP), and *Chlamydia pneumoniae* (Cpn).

Of the 232 HCoV-positive cases, 142 (61.2%) involved single-subtype infections, and the remaining 90 (38.8%) showed co-infections with multiple pathogens (Fig. 3A). Six cases represented co-infections among the four subtypes, including two cases of HCoV-229E + HCoV-NL63, three cases of HCoV-NL63 + HCoV-OC43, and one case of HCoV-OC43 + HCoV-HKU1 co-infections (Fig. 3A). In addition to inter-subtype co-infections, several HCoV-positive cases exhibited co-infections with other respiratory pathogens, including viral co-infections (excluding HCoVs), bacterial co-infections, and combined viral-bacterial co-infections (Fig. 3A). Notably, HCoVs showed higher co-detection frequencies with bacteria than with other viruses. The top three frequently co-detected bacterial pathogens were *Streptococcus pneumoniae*, *Haemophilus influenzae*, and *Klebsiella pneumoniae*, while HMPV, rhinovirus, and influenza viruses were the predominant viral co-pathogens (Fig. 3A-3B and S4 Table). *Streptococcus pneumoniae*, *Haemophilus influenzae*, *Klebsiella pneumoniae*, HCoVs, rhinovirus, and HMPV were also the common co-infecting pathogens with other frequently detected respiratory pathogens (S5 Table). Additionally, *Chlamydia pneumoniae* (one case), *Mycoplasma pneumoniae* (one case), and *Chlamydia psittaci* (one case) were identified in HCoV-positive co-infections (Fig. 3A).

### 3.4 Comparison of circulation trends with common respiratory viruses

**Fig. 4:**
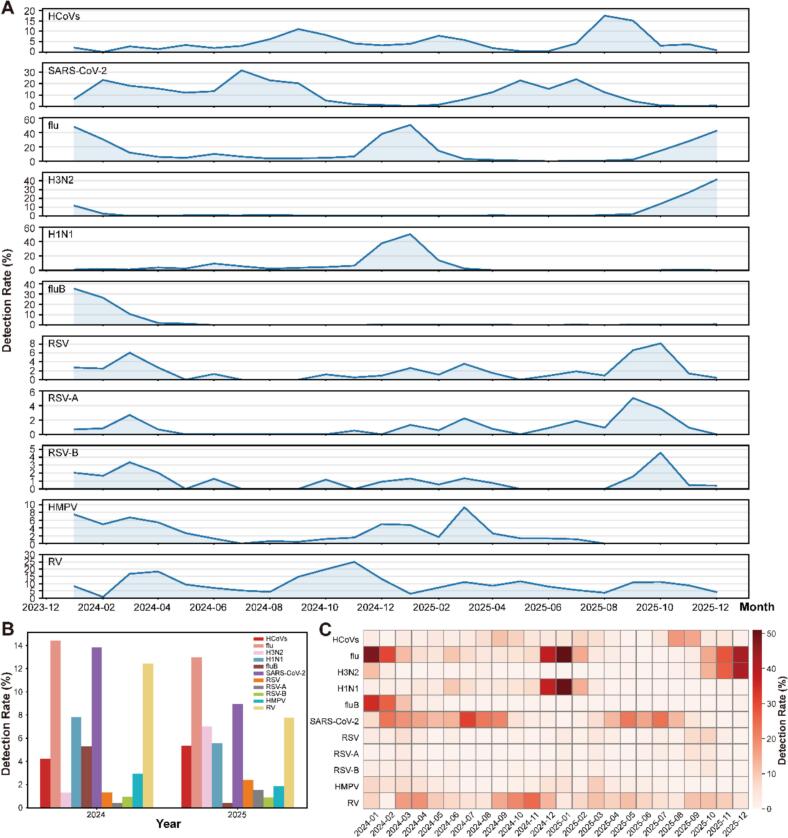
Comparison of epidemiological trends between human coronaviruses and several common respiratory viruses in Jing'an District, Shanghai, 2024–2025. A. Monthly detection rates of HCoVs and several common respiratory viruses. Each panel represents an individual pathogen, and all panels share a common time axis. B. Annual overall detection rates of HCoVs and several common respiratory viruses. C. Heatmap of monthly detection rates of HCoVs and several common respiratory viruses. Color gradients indicate the magnitude of detection rates, with darker red colors representing higher values. Several common respiratory pathogens were included: human coronaviruses (HCoVs), Severe Acute Respiratory Syndrome Coronavirus-2 (SARS-CoV-2), influenza A virus (flu), influenza A virus (H3N2), influenza A virus (H1N1), influenza B virus (fluB), respiratory syncytial virus (RSV), respiratory syncytial virus A (RSV-A), respiratory syncytial virus B (RSV-B), human metapneumovirus (HMPV), and rhinovirus (RV). (For interpretation of the references to color in this figure legend, the reader is referred to the web version of this article.)

Among the 4758 cases, the overall infection rates of the six common respiratory viruses were as follows: influenza viruses (13.6%), SARS-CoV-2 (11.0%), rhinovirus (9.8%), HCoVs (4.9%), HMPV (2.3%), and RSV (2.0%) (Table 1). The prevalence of influenza viruses, SARS-CoV-2, and rhinovirus was significantly higher than that of HCoVs, HMPV, and RSV (Fig. 4A-4C). Alternating and co-circulation patterns were observed among these viruses. Influenza activity peaked during the winter months, followed by increased circulation of HMPV and RSV in winter and early spring. HCoVs showed higher activity during summer and autumn. SARS-CoV-2 exhibited a cross-seasonal bimodal distribution with year-round detection. Rhinovirus demonstrated two seasonal peaks in spring and autumn (March–April and October–November). Co-circulation of multiple viruses was observed during several transitional periods, particularly in January and December (Fig. 4A).

Furthermore, detailed analysis revealed complex temporal variations within individual viral subtypes. As noted above, alternating circulation patterns were observed among different HCoVs subtypes. Similar alternation was also evident among influenza virus subtypes: influenza B predominated during winter and early spring in 2024; H1N1 was dominant in late winter of 2024; whereas H3N2 showed increased activity during late winter in 2025. In contrast, the two RSV subtypes exhibited highly overlapping epidemic cycles, indicating a co-circulation pattern (Fig. 4A).

## 4 Discussion

This study conducted a comprehensive analysis of the 2024–2025 surveillance data from Jing'an District, Shanghai, to characterize the epidemiology of HCoVs among patients with acute respiratory infections. During the study period, HCoVs exhibited subtype-specific seasonal predominance and marked age stratification, suggesting that prevention and control strategies should prioritize high-risk populations, such as children and the elderly, as well as periods of increased viral activity.

Through comprehensive analysis across temporal and population dimensions, this study demonstrates that different HCoV subtypes in acute respiratory infection cases in Jing'an District, Shanghai, exhibited distinct seasonal patterns and age-specific preferences. HCoV-NL63 peaked in late summer to early autumn, HCoV-OC43 in autumn and winter, and HCoV-HKU1 in winter and spring. These findings align broadly with reports from regions of similar climate (*e.g.*, Taiwan (Wu et al., 2008), Hong Kong (Chiu et al., 2005), and Guangzhou (Zhang et al., 2018)). In addition, similar surveillance studies conducted in Shanghai (Kong et al., 2021) and in Pudong New District, Shanghai (Zhang et al., 2024), provide some supporting evidence for our findings. Our observations are based on only two years of data and cannot confirm long-term circulation patterns. Multi-year, continuous surveillance is needed to clarify seasonal trends and potential driving factors, including climatic factors (temperature, humidity, sunshine duration, and wind speed) and population activities (*e.g.*, school reopenings, indoor gatherings) (Earn et al., 2012; Moriyama et al., 2020).

Regarding age distribution, this study identified children under 14 years and adults over 65 years as susceptible populations for HCoVs, with notable variations in subtype composition across age groups. Specifically, HCoV-NL63 was more prevalent in the 0–4 and 5–14 age groups; HCoV-OC43 predominated in both the 0–4 and ≥ 65 age groups. This age-specific subtype preference is supported by previous studies. Dijkman et al. (Dijkman et al., 2012) reported higher infection rates of HCoV-NL63 and HCoV-OC43 during childhood, and a review by Wilson et al. (Wilson et al., 2024) noted that median detection rates of HCoV-OC43 and HCoV-NL63 were higher in children compared with adults. A study (Han et al., 2023) from China similarly highlights children, particularly infants and young children, as a high-risk population for HCoVs infections. Additionally, Saletti et al. (Saletti et al., 2020) observed that elderly individuals exhibit reduced frequencies of subtype-specific T-cell responses to HCoV-OC43 compared with younger adults, potentially affecting susceptibility. Based on the above, this study infers that the biological mechanisms underlying age-stratified susceptibility may be twofold: children possess an immature immune system with limited prior exposure and underdeveloped virus-specific immunity, while elderly individuals may be more vulnerable to certain subtypes, such as HCoV-OC43, due to immunosenescence, waning antibody titres from prior exposure, and diminished mucosal defense functions.

This study found a certain degree of co-infections between HCoVs and several common respiratory viruses, such as influenza viruses, SARS-CoV-2, and RSV, alongside alternating and co-circulating patterns in their epidemiological trends. Previous studies (Friedman et al., 2018; Gaunt et al., 2010) have shown that co-infections with HCoVs and other respiratory pathogens are relatively common, where two or more pathogens are concurrently detected. Furthermore, this study observed that for common respiratory viruses, the rates of virus-bacteria co-infections were generally higher than those of virus-virus co-infections. Viral infections can compromise epithelial barriers and immune defenses, facilitating secondary bacterial infections (Bellinghausen et al., 2016; Bosch et al., 2013; Man et al., 2017; Watson et al., 2006). These findings highlight the importance of considering multi-pathogen co-infections in surveillance, clinical management, and prevention strategies.

However, the study was limited to four sentinel hospitals in Jing'an District, Shanghai, with incomplete clinical data, restricting in-depth analysis of co-infection outcomes. The two-year study period highlights the need for long-term surveillance. Future multicenter studies incorporating viral genotyping and host immunological analyses could improve understanding of post-pandemic HCoVs evolution and support integrated control strategies for respiratory pathogens.

## 5 Conclusions

The study indicates that during 2024–2025 in Jing'an District, Shanghai, different HCoV subtypes dominated in distinct seasons and showed pronounced age-specific preferences in patients with acute respiratory infections. Based on these observations and prior surveillance experience in China, we recommend continued integrated monitoring of HCoVs with other respiratory pathogens, prioritizing high-risk populations to improve laboratory efficiency and optimize medical resource allocation. Multi-pathogen surveillance combined with molecular epidemiology could further clarify interactions and cross-immunity among pathogens, informing stratified prevention and predictive modeling.

## Abbreviations

**Table:** 

HCoVs	human coronaviruses
HCoV-NL63	human coronavirus NL63
HCoV-229E	human coronavirus 229E
HCoV-OC43	human coronavirus OC43
HCoV-HKU1	human coronavirus HKU1
flu	influenza viruses
RSV	respiratory syncytial virus
RV	rhinovirus
ILI	influenza-like illness
SARI	severe acute respiratory infection
HPIV	human parainfluenza viruses
ADV	adenovirus
EV	enterovirus
HMPV	human metapneumovirus
HBoV	human bocavirus
Spn	*Streptococcus pneumoniae*
Hi	*Haemophilus influenzae*
KP	*Klebsiella pneumoniae*
MP	*Mycoplasma pneumoniae*
Cpn	*Chlamydia pneumoniae*
GAS	*Group A Streptococcus*.

## CRediT authorship contribution statement

**Qi Shen:** Writing – original draft, Resources, Investigation, Data curation. **Shuiping Lu:** Writing – original draft, Visualization, Methodology, Formal analysis. **Qingyuan Xu:** Writing – review & editing, Validation, Resources, Investigation. **Mengting Tang:** Writing – review & editing, Validation, Resources, Investigation. **Yi Li:** Writing – review & editing, Supervision, Funding acquisition, Conceptualization. **Bing Shen:** Writing – review & editing, Validation, Resources, Investigation. **Mingyi Cai:** Writing – review & editing, Supervision, Resources, Investigation, Conceptualization. **Chenglong Xiong:** Writing – review & editing, Supervision, Project administration, Conceptualization.

## Ethics approval and consent to participate

This surveillance was conducted as part of routine public health monitoring activities in Shanghai. The study protocol was reviewed and approved by the Medical Ethics Committee of Shanghai Jing'an District Central Hospital following submission by the Jing'an District Center for Disease Control and Prevention, Shanghai. Informed consent from individual participants was not required, as only anonymized data were used. All patient data were de-identified before analysis to ensure confidentiality. The study adhered to the ethical principles of the Declaration of Helsinki.

## Funding

This research was funded by the Jing'an District Three-Year Action Plan for Strengthening the Public Health System (JAGW2023201 to Yi Li; JAGW2023110 to Yi Li). The funder had no role in study design, data collection and analysis, decision to publish, or preparation of the manuscript.

## Declaration of competing interest

The authors declare that they have no known competing financial interests or personal relationships that could have appeared to influence the work reported in this paper.

## Data Availability

Data will be made available on request.

## Appendix A Supplementary data

Supplementary material S1 Fig Overview of respiratory pathogen detections in Jing'an District, Shanghai, 2024-2025. S1 Table Age and gender distribution of human coronaviruses in Jing'an District, Shanghai, 2024-2025. S2 Table Monthly detection of human coronaviruses positive cases in Jing'an District, Shanghai, 2024-2025. S3 Table Seasonal detection of human coronaviruses positive cases in Jing'an District, Shanghai, 2024-2025. S4 Table Co-detected rates of human coronaviruses with other respiratory pathogens in Jing'an District, Shanghai, 2024-2025. S5 Table Co-detected rates of several common respiratory viruses with other respiratory pathogens in Jing'an District, Shanghai, 2024-2025. Image 1
