# Supplementary material for: Epidemiological characteristics of human coronaviruses among populations with acute respiratory infections: surveillance data from jing'an district, Shanghai, 2024–2025
Source: Prev Med Rep. 2026 Feb 4;62:103401. doi: 10.1016/j.pmedr.2026.103401 (PMC12907243; doi:10.1016/j.pmedr.2026.103401)
Supplement: Supplementary file 1 — Supplementary material S1 Fig Overview of respiratory pathogen detections in Jing'an District, Shanghai, 2024-2025. S1 Table Age and gender distribution of human coronaviruses in Jing'an District, Shanghai, 2024-2025. S2 Table Monthly detection of human coronaviruses positive cases in Jing'an District, Shanghai, 2024-2025. S3 Table Seasonal detection of human coronaviruses positive cases in Jing'an District, Shanghai, 2024-2025. S4 Table Co-detected rates of human coronaviruses with other respiratory pathogens in Jing'an District, Shanghai, 2024-2025. S5 Table Co-detected rates of several common respiratory viruses with other respiratory pathogens in Jing'an District, Shanghai, 2024-2025. [file mmc1.docx]

**Appendix A. Supporting information**

**Epidemiological Characteristics of Human Coronaviruses Among** **Populations with** **Acute Respiratory Infections: Surveillance Data from** **Jing'an District, Shanghai, 2024-2025**

**Qi Shen^1¶^, Shuiping Lu^2¶^, Qingyuan Xu^1^, Mengting Tang^1^, Yi Li^1^, Bing Shen^1^, Mingyi Cai^1*^, Chenglong Xiong^2*^**

**^1^** Shanghai Jing'an District Center for Disease Control and Prevention (Shanghai Jing'an District Health Supervision Institute), Shanghai 200072, China

**^2^** School of Public Health, Fudan University, Key Lab of Public Health Safety, Ministry of Education, Shanghai 200433, China

**^¶^** These authors contributed equally to this work.

**^*^** Corresponding author: xiongchenglong@fudan.edu.cn (C. X.), [caimingyi@jingancdc.net](mailto:caimingyi@jingancdc.net) (M. C.)

This file includes:

S1 Fig

S1 to S5 Tables


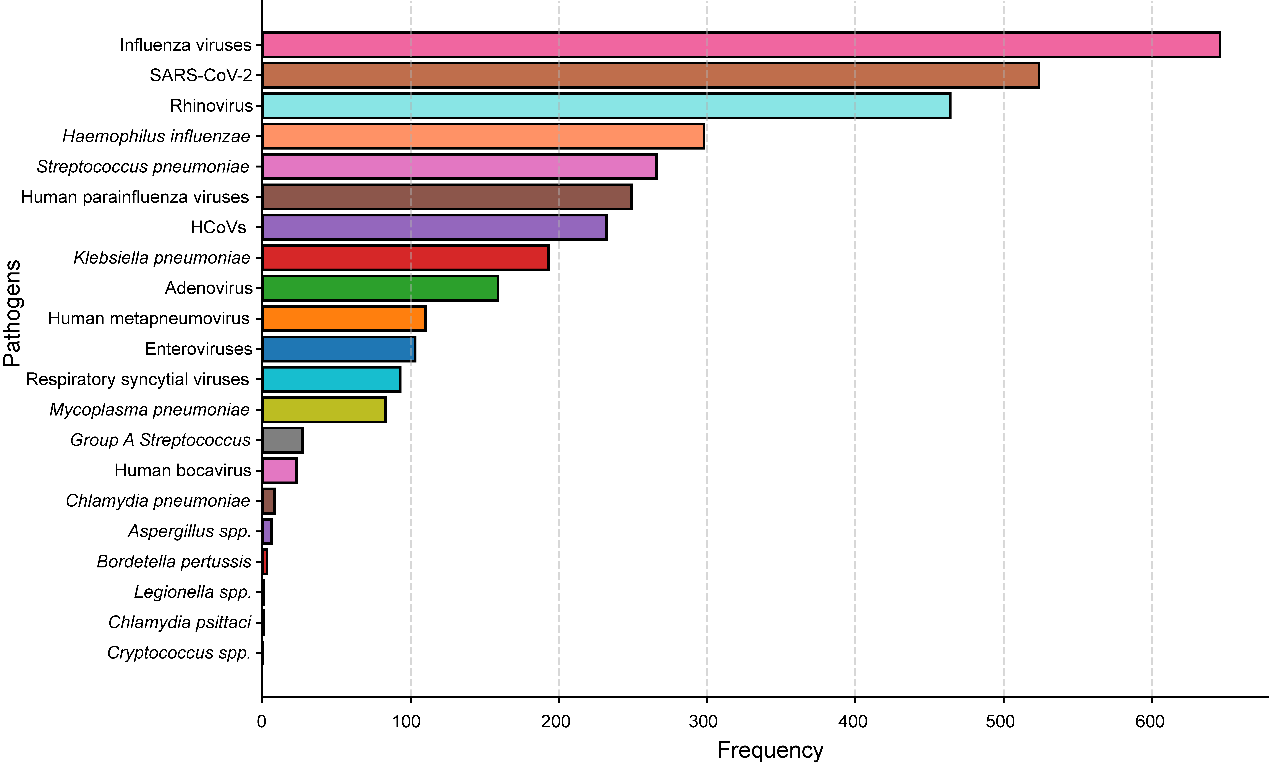


**S1 Fig. Overview of respiratory pathogen detections in Jing'an District, Shanghai, 2024-2025**. Influenza viruses: influenza A virus (H1N1, H3N2) and influenza B virus. Severe Acute Respiratory Syndrome Coronavirus-2 (SARS-CoV-2). Human parainfluenza viruses (HPIV): HPIV1, HPIV2, HPIV3, HPIV4. Respiratory syncytial viruses (RSV): RSV-A, RSV-B. Human coronaviruses (HCoVs): HCoV-229E, HCoV-HKU1, HCoV-OC43, HCoV-NL63. *Cryptococcus spp.* was not detected.

**S1 Table. Age and gender distribution of human coronaviruses in Jing'an District, Shanghai, 2024-2025**

| Variables | Total (N = 4758) | HCoVs (N = 232) | | HCoV-229E (N = 20) | | HCoV-NL63 (N = 125) | | HCoV-OC43 (N = 53) | | HCoV-HKU1 (N = 40) | |
| --- | --- | --- | --- | --- | --- | --- | --- | --- | --- | --- | --- |
|  |  | No.positive | Rate (%)^a^ | No.positive | Rate (%) | No.positive | Rate (%) | No.positive | Rate (%) | No.positive | Rate (%) |
| Gender |  | χ² = 5.66, P = 0.02^b^ | | χ² = 0.027, P = 0.87 | | χ² = 3.00, P = 0.08 | | χ² = 1.12, P = 0.29 | | χ² = 1.41, P = 0.24 | |
| Male | 2228 | 91 | 4.1 | 9 | 0.4 | 49 | 2.2 | 21 | 0.9 | 15 | 0.7 |
| Female | 2530 | 141 | 5.6 | 11 | 0.4 | 76 | 3.0 | 32 | 1.3 | 25 | 1.0 |
| Age groups, year |  | χ² = 32.79, P < 0.001 | | Fisher, P = 0.62^c^ | | χ² = 56.33, P < 0.001 | | Fisher, P = 0.002 | | Fisher, P = 0.62 | |
| 0-4 | 562 | 51 | 9.1 | 4 | 0.7 | 33 | 5.9 | 12 | 2.1 | 5 | 0.9 |
| 5-14 | 1481 | 82 | 5.5 | 5 | 0.3 | 59 | 4.0 | 5 | 0.3 | 15 | 1.0 |
| 15-24 | 247 | 9 | 3.6 | 0 | 0.0 | 4 | 1.6 | 2 | 0.8 | 4 | 1.6 |
| 25-44 | 1116 | 46 | 4.1 | 7 | 0.6 | 17 | 1.5 | 15 | 1.3 | 7 | 0.6 |
| 45-64 | 513 | 15 | 2.9 | 1 | 0.2 | 6 | 1.2 | 5 | 1.0 | 3 | 0.6 |
| ≥65 | 839 | 29 | 3.5 | 3 | 0.4 | 6 | 0.7 | 14 | 1.7 | 6 | 0.7 |

^a^ Detection rate is calculated as the number of positive samples in a category divided by the total samples collected in that category.

^b^ Pearson's chi-square test.

^c^ Fisher's exact test.

**S2 Table.** **Monthly detection of human coronaviruses positive cases in Jing'an District, Shanghai, 2024-2025**

| Month | Total | HCoVs | | HCoV-229E | | HCoV-NL63 | | HCoV-OC43 | | HCoV-HKU1 | |
| --- | --- | --- | --- | --- | --- | --- | --- | --- | --- | --- | --- |
|  |  | No.positive | Rate (%)^a^ | No.positive | Rate (%) | No.positive | Rate (%) | No.positive | Rate (%) | No.positive | Rate (%) |
| 2024 | | | | | | | | | | | |
| Jan | 148 | 3 | 2.0 | 3 | 2.0 | 0 | 0.0 | 0 | 0.0 | 0 | 0.0 |
| Feb | 121 | 0 | 0.0 | 0 | 0.0 | 0 | 0.0 | 0 | 0.0 | 0 | 0.0 |
| Mar | 149 | 4 | 2.7 | 0 | 0.0 | 0 | 0.0 | 2 | 1.3 | 3 | 2.0 |
| Apr | 147 | 2 | 1.4 | 0 | 0.0 | 1 | 0.7 | 0 | 0.0 | 1 | 0.7 |
| May | 149 | 5 | 3.4 | 1 | 0.7 | 0 | 0.0 | 1 | 0.7 | 3 | 2.0 |
| Jun | 157 | 3 | 1.9 | 1 | 0.6 | 1 | 0.6 | 0 | 0.0 | 1 | 0.6 |
| Jul | 171 | 5 | 2.9 | 0 | 0.0 | 3 | 1.8 | 2 | 1.2 | 0 | 0.0 |
| Aug | 162 | 10 | 6.2 | 0 | 0.0 | 8 | 4.9 | 3 | 1.9 | 0 | 0.0 |
| Sept | 217 | 24 | 11.1 | 0 | 0.0 | 22 | 10.1 | 2 | 0.9 | 0 | 0.0 |
| Oct | 171 | 14 | 8.2 | 1 | 0.6 | 4 | 2.3 | 8 | 4.7 | 1 | 0.6 |
| Nov | 196 | 8 | 4.1 | 0 | 0.0 | 3 | 1.5 | 5 | 2.6 | 1 | 0.5 |
| Dec | 220 | 7 | 3.2 | 0 | 0.0 | 1 | 0.5 | 5 | 2.3 | 1 | 0.5 |
| 2025 | | | | | | | | | | | |
| Jan | 230 | 9 | 3.9 | 0 | 0.0 | 2 | 0.9 | 4 | 1.7 | 4 | 1.7 |
| Feb | 179 | 14 | 7.8 | 1 | 0.6 | 0 | 0.0 | 2 | 1.1 | 11 | 6.1 |
| Mar | 225 | 13 | 5.8 | 0 | 0.0 | 1 | 0.4 | 2 | 0.9 | 10 | 4.4 |
| Apr | 270 | 5 | 1.9 | 0 | 0.0 | 0 | 0.0 | 3 | 1.1 | 2 | 0.7 |
| May | 224 | 1 | 0.4 | 1 | 0.4 | 0 | 0.0 | 0 | 0.0 | 0 | 0.0 |
| Jun | 227 | 1 | 0.4 | 0 | 0.0 | 0 | 0.0 | 1 | 0.4 | 0 | 0.0 |
| Jul | 269 | 11 | 4.1 | 3 | 1.1 | 7 | 2.6 | 0 | 0.0 | 1 | 0.4 |
| Aug | 217 | 38 | 17.5 | 3 | 1.4 | 33 | 15.2 | 1 | 0.5 | 1 | 0.5 |
| Sept | 258 | 39 | 15.1 | 4 | 1.6 | 33 | 12.8 | 3 | 1.2 | 0 | 0.0 |
| Oct | 197 | 6 | 3.0 | 1 | 0.5 | 5 | 2.5 | 1 | 0.5 | 0 | 0.0 |
| Nov | 217 | 8 | 3.7 | 1 | 0.5 | 1 | 0.5 | 6 | 2.8 | 0 | 0.0 |
| Dec | 237 | 2 | 0.8 | 0 | 0.0 | 0 | 0.0 | 2 | 0.8 | 0 | 0.0 |

^a^ Monthly detection rate is calculated as the number of positive samples in a month divided by the total samples collected in that month.

**S3 Table. Seasonal detection of human coronaviruses positive cases in Jing'an District, Shanghai, 2024-2025**

| Season  2024-2025 | Total | HCoVs | | HCoV-229E | | HCoV-NL63 | | HCoV-OC43 | | HCoV-HKU1 | |
| --- | --- | --- | --- | --- | --- | --- | --- | --- | --- | --- | --- |
|  |  | No.positive | Rate (%)^a^ | No.positive | Rate (%) | No.positive | Rate (%) | No.positive | Rate (%) | No.positive | Rate (%) |
| Winter (Jan-Feb) | 269 | 3 | 1.1 | 3 | 1.1 | 0 | 0.0 | 0 | 0.0 | 0 | 0.0 |
| Spring | 445 | 11 | 2.5 | 1 | 0.2 | 1 | 0.2 | 3 | 0.7 | 7 | 1.6 |
| Summer | 490 | 18 | 3.7 | 1 | 0.2 | 12 | 2.4 | 5 | 1.0 | 1 | 0.2 |
| Autumn | 584 | 46 | 7.9 | 1 | 0.2 | 29 | 5.0 | 15 | 2.6 | 2 | 0.3 |
| Winter | 629 | 30 | 4.8 | 1 | 0.2 | 3 | 0.5 | 11 | 1.7 | 16 | 2.5 |
| Spring | 719 | 19 | 2.6 | 1 | 0.1 | 1 | 0.1 | 5 | 0.7 | 12 | 1.7 |
| Summer | 713 | 50 | 7.0 | 6 | 0.8 | 40 | 5.6 | 2 | 0.3 | 2 | 0.3 |
| Autumn | 672 | 53 | 7.9 | 6 | 0.9 | 39 | 5.8 | 10 | 1.5 | 0 | 0.0 |
| Winter (Dec) | 237 | 2 | 0.8 | 0 | 0.0 | 0 | 0.0 | 2 | 0.8 | 0 | 0.0 |

^a^ Seasonal detection rate is calculated as the number of positive samples in a seasonal period divided by the total samples collected in that seasonal period.

**S4 Table. Co-detected rates of human coronaviruses with other respiratory pathogens in Jing'an District, Shanghai, 2024-2025**

| Co-detected rates* (%) | HCoVs | HCoV-229E | HCoV-HKU1 | HCoV-OC43 | HCoV-NL63 |
| --- | --- | --- | --- | --- | --- |
| H1N1 | 1.9 | 0.0 | 1.4 | 1.1 | 0.5 |
| H3N2 | 0.7 | 0.4 | 0.0 | 0.4 | 0.3 |
| fluB | 0.3 | 0.7 | 0.0 | 0.0 | 0.0 |
| RV | 2.1 | 0.0 | 0.4 | 0.8 | 1.6 |
| SARS-CoV-2 | 1.2 | 0.0 | 0.4 | 0.5 | 0.6 |
| HMPV | 2.1 | 0.0 | 2.0 | 1.9 | 0.9 |
| RSV-A | 0.7 | 1.4 | 0.0 | 1.0 | 0.6 |
| RSV-B | 0.4 | 1.6 | 0.0 | 0.0 | 0.6 |
| ADV | 0.8 | 0.6 | 1.0 | 0.0 | 0.0 |
| EV | 0.6 | 0.0 | 0.0 | 0.6 | 0.4 |
| HBoV | 0.8 | 0.0 | 0.0 | 1.3 | 0.7 |
| HPIV1 | 0.3 | 0.0 | 0.0 | 0.8 | 0.0 |
| HPIV3 | 0.3 | 0.0 | 0.0 | 0.0 | 0.5 |
| Spn | 5.1 | 0.7 | 1.0 | 0.9 | 4.5 |
| Hi | 4.7 | 0.0 | 1.2 | 2.0 | 3.7 |
| KP | 2.9 | 0.0 | 0.4 | 1.7 | 2.3 |
| GAS | 0.8 | 0.0 | 1.5 | 0.0 | 0.7 |
| Cpn | 0.4 | 0.0 | 0.0 | 0.0 | 0.8 |
| MP | 0.3 | 0.0 | 0.0 | 0.7 | 0.0 |
| CP | 0.4 | 0.0 | 0.0 | 1.9 | 0.0 |

* Co-detected rates were calculated pairwise. For pathogens "X" and "Y", the numerator was the number of patients in whom "X" and "Y" were co-detected. The denominator was the total number of patients tested "X" and "Y". Several respiratory pathogens were included: human coronaviruses (HCoVs), human coronavirus NL63 (HCoV-NL63), human coronavirus 229E (HCoV-229E), human coronavirus OC43 (HCoV-OC43), human coronavirus HKU1 (HCoV-HKU1), rhinovirus (RV), adenovirus (ADV), enterovirus (EV), human metapneumovirus (HMPV), respiratory syncytial virus A (RSV-A), respiratory syncytial virus B (RSV-B), Severe Acute Respiratory Syndrome Coronavirus-2 (SARS-CoV-2), influenza A virus (H1N1), influenza A virus (H3N2), influenza B virus (fluB), human parainfluenza virus 1 (HPIV1), human parainfluenza virus 3 (HPIV3), human bocavirus (HBoV), *Streptococcus pneumoniae* (Spn), *Haemophilus influenzae* (Hi), *Klebsiella pneumoniae* (KP), *Group A Streptococcus* (GAS), *Mycoplasma pneumoniae* (MP), *Chlamydia psittaci* (CP), and *Chlamydia pneumoniae* (Cpn).

**S5 Table. Co-detected rates of several common respiratory viruses with other respiratory pathogens in Jing'an District, Shanghai, 2024-2025**

| Pathogen | Virus (top3^a^) | Co-detected rates* (%) | Other pathogens (top3) | Co-detected rates (%) |
| --- | --- | --- | --- | --- |
| SARS-CoV-2 | HCoVs | 1.2 | Spn | 2.7 |
|  | RV | 0.8 | Hi | 2.4 |
|  | EV | 0.6 | KP | 1.7 |
| H1N1 | RV | 2.5 | Hi | 3.8 |
|  | HCoVs | 1.9 | Spn | 2.5 |
|  | EV | 0.5 | KP | 0.4 |
| H3N2 | HCoVs | 0.7 | Hi | 3.2 |
|  | RV | 0.6 | Spn | 2.8 |
|  | HBoV | 0.4 | KP | 0.7 |
| fluB | RV | 1.0 | Spn | 2.4 |
|  | HMPV | 0.9 | Hi | 0.2 |
|  | HCoVs | 0.3 | /^b^ | / |
| RSV-A | HCoVs | 0.7 | Cpn | 1.8 |
|  | HMPV | 0.6 | Spn | 1.6 |
|  | ADV | 0.5 | Hi | 1.5 |
| RSV-B | HMPV | 0.7 | MP | 2.4 |
|  | ADV | 0.5 | Spn | 1.3 |
|  | RV | 0.4 | KP | 1.3 |
|  | HCoVs | 0.4 |  |  |
| RV | EV | 5.2 | Spn | 6.9 |
|  | ADV | 3.0 | Hi | 5.4 |
|  | H1N1 | 2.5 | MP | 2.1 |
|  | HCoVs | 2.1 |  |  |
| HMPV | HCoVs | 2.1 | Spn | 2.2 |
|  | RV | 1.2 | Hi | 1.5 |
|  | ADV | 1.1 | MP | 1.1 |

* Co-detected rates were calculated pairwise. For pathogens "X" and "Y", the numerator was the number of patients in whom "X" and "Y" were co-detected. The denominator was the total number of patients tested "X" and "Y".

^a^ The top three viruses or other pathogens exhibiting the highest co-detection rates are presented.

^b^ In this study, co-infections involving FluB were observed exclusively with two bacterial pathogens: *Streptococcus pneumoniae* (Spn) and *Haemophilus influenzae* (Hi).

Several respiratory pathogens were included: human coronaviruses (HCoVs), rhinovirus (RV), adenovirus (ADV), enterovirus (EV), human metapneumovirus (HMPV), respiratory syncytial virus A (RSV-A), respiratory syncytial virus B (RSV-B), Severe Acute Respiratory Syndrome Coronavirus-2 (SARS-CoV-2), influenza A virus (H1N1), influenza A virus (H3N2), influenza B virus (fluB), human bocavirus (HBoV), *Streptococcus pneumoniae* (Spn), *Haemophilus influenzae* (Hi), *Klebsiella pneumoniae* (KP), *Mycoplasma pneumoniae* (MP), and *Chlamydia pneumoniae* (Cpn).
